# The Many Faces of Enteric Fever: A Case Report on Septic Shock With Tachyarrhythmias, Icterus With Isolated Thrombocytopenia, and Enterocolitis With Leukopenia

**DOI:** 10.7759/cureus.92543

**Published:** 2025-09-17

**Authors:** Sujata J Khatal, Bryan Koithara, Aamir Khatri

**Affiliations:** 1 Department of Internal Medicine, Bharati Vidyapeeth (Deemed to Be University) Medical College, Pune, IND

**Keywords:** enterocolitis, isolated thrombocytopenia, leukopenia - low white cell count, salmonella bacteremia, salmonella complications, sepsis and septic shock, tachyarrhythmias, typhoidal strains of salmonella

## Abstract

Enteric fever is a serious systemic infection that collectively encompasses typhoid and paratyphoid fevers, caused by *Salmonella enterica* serovars Typhi and Paratyphi, respectively. It is a major public health concern worldwide and may present a complex clinical challenge to physicians in endemic regions. We present a report of three cases highlighting atypical presentations and complications of enteric fever. The first case reports on a 55-year-old man who presented with septic shock complicated by sequential tachyarrhythmias, despite a structurally normal heart and normal serum electrolyte levels, requiring chemical cardioversion. An infection by Salmonella Paratyphi B was confirmed by blood and stool cultures. Subsequent targeted antimicrobial therapy resulted in complete clearance of the infection. Our second case recounts the clinical course of a 25-year-old male patient who presented with sustained fever, icterus, and severe thrombocytopenia. The tropical fever serology panel was noncontributory, but blood culture confirmed Salmonella Typhi infection. Targeted therapy with intravenous ceftriaxone led to complete recovery. The third case presents a 64-year-old female patient with diabetes and hypertension, who presented with an acute abdomen associated with septic shock. Abdominal imaging was performed, which suggested enterocolitis. Empirical antibiotic therapy was met with a modest response, but her clinical course was complicated by worsening leukopenia. The isolation of Salmonella Paratyphi B on blood culture allowed the timely optimization of antibiotic therapy, leading to immune reconstitution and, ultimately, complete clinical recovery. The present report on enteric fever aims to highlight its wide spectrum and uncommon complications observed during its course. Treating clinicians will benefit from maintaining a high index of suspicion for unusual presenting features and culture-guided therapy, especially in endemic regions, to optimize and expedite clinical outcomes.

## Introduction

Enteric fever, caused by *S. enterica* serovar Typhi and *S. enterica* serovar Paratyphi, is a febrile multisystemic infection endemic to low- and middle-income countries. Its endemicity in these regions is attributed to a lack of access to safe drinking water, inadequate hygiene practices, and poor vaccine coverage [1]. Salmonella are gram-negative, obligate anaerobes belonging to the family Enterobacteriaceae. While several species of Salmonella are known to infect animals, in addition to humans, the *S. enterica* species is exclusively pathogenic to humans. The source of human infection is typically contaminated food or water. After its ingestion, Salmonella reaches the small intestines and penetrates the gut epithelium. After a median incubation period of 5-12 days, which coincides with a transient phase of primary bacteremia, colonization of the lymphoid tissue within Peyer’s patch and the gall bladder occurs [2]. The onset of febrile illness and gastrointestinal symptoms heralds an established infection, following which persistent secondary bacteremia and dissemination to the liver, spleen, and bone marrow can occur, potentially resulting in serious systemic manifestations [3]. The classical presentation of enteric fever is sustained fever, malaise, and abdominal discomfort, accompanied by varying degrees of nonspecific gastrointestinal manifestations. Unusual systemic complications can occur, usually after the second to third week of illness [4]. This can lead to diagnostic misinterpretation and inappropriate management. A diagnosis is best made by isolating Salmonella from blood and bone marrow, and less frequently from stool and urine, depending on the phase of the illness [5].

The present report on enteric fever documents the clinical course of three patients, presenting with uncommon complications such as septic shock with sequential tachyarrhythmias, icterus with isolated thrombocytopenia, and enterocolitis complicated by leukopenia.

## Case presentation

Case 1

A 55-year-old male patient presented to our emergency room with a history of fever for three days, associated with generalized weakness, abdominal pain, and bloating. He lacked any known comorbidities. A general physical examination revealed a pulse of 116 beats/minute, a blood pressure of 90/60 mmHg, and cold, clammy extremities. He, however, lacked any signs of dehydration, and his systemic examination revealed a diffusely tender abdomen. Suspecting septic shock with an abdominal focus, blood, stool, and urine cultures were sent, and he was started on empirical intravenous ceftriaxone, metronidazole, and vasopressors. Preliminary laboratory results showed leukocytosis (total leukocyte count of 13,400 per μL), associated with elevated serum procalcitonin levels (8.66 ng/mL) and raised venous lactate (6.2 mEq/L), confirming sepsis. His laboratory results also showed nonoliguric acute kidney injury (AKI; serum creatinine of 4.33 mg/dL) and elevated levels of serum aspartate transaminase (141 U/L). A point-of-care ultrasound (POCUS) of his abdomen showed fatty infiltration of the liver. His hospital course was further complicated by two episodes of paroxysmal supraventricular tachycardia followed by an episode of sustained atrial fibrillation, requiring chemical cardioversion to restore sinus rhythm. The cause of sequential tachyarrhythmias was not forthcoming, as his serum electrolyte levels were within normal limits, and a two-dimensional echocardiogram showed a structurally and functionally normal heart. Urine culture did not yield any pathogens, but his blood and stool cultures did isolate Salmonella Paratyphi B. Antibiotic therapy was subsequently optimized by the withdrawal of metronidazole and escalating the dose of ceftriaxone to 2 g, twice-daily, following which complete clinical resolution was achieved.

Case 2

A 25-year-old male patient presented to our outpatient department with a history of fever for eight days, accompanied by diarrhea and abdominal pain. A general physical examination revealed icterus and bilateral, nonpitting, pedal edema. A per-abdominal exam was performed, which revealed only mild epigastric tenderness on deep palpation. His remaining systemic examination findings were unremarkable. Initial laboratory test results confirmed direct hyperbilirubinemia with elevated levels of serum total and direct bilirubin (8.8 and 7.04 mg/dL, respectively). Severe thrombocytopenia was also present (platelet count of 26,000/mm^3^) despite normal serum hemoglobin levels and adequate total leukocyte count. His presenting features, accompanied by direct hyperbilirubinemia and severe isolated thrombocytopenia, made us consider a tropical illness. Thus, a tropical fever polymerase chain reaction panel was ordered, which included serological markers for dengue, chikungunya, leptospirosis, rickettsial infections, and malaria, which was negative. HIV and Hepatitis B and C serologies were negative as well. A POCUS of his abdomen showed hepatomegaly (17 cm) and pseudothickening of the gallbladder wall. While enteric fever does not classically present with icterus, his predominant gastrointestinal findings coupled with early isolated thrombocytopenia allowed us to maintain a high index of suspicion for typhoid, despite negative serology. Our suspicions were confirmed by an isolate of Salmonella Typhi on blood culture. He was started on intravenous ceftriaxone at a dose of 2 g, twice daily. A good clinical response was indicated by resolution of thrombocytopenia and direct hyperbilirubinemia accompanied by symptom clearance.

Case 3

**Figure 1:**
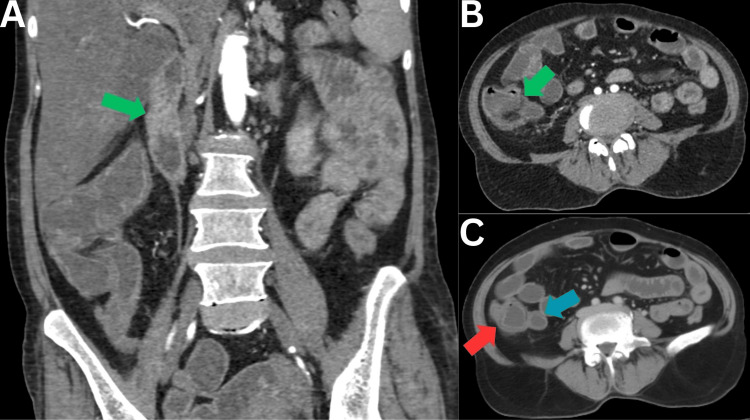
Contrast-enhanced CT coronal (A) and axial (B,C) sections of the abdomen and pelvis, showing edematous wall thickening involving the ileocecal junction (green arrows), cecum (red arrow), and the terminal ileum (blue arrow)

A 64-year-old female patient, known diabetic and hypertensive, presented to our emergency room with a history of fever for two days, accompanied by multiple episodes of diarrhea. On initial evaluation, she appeared drowsy and had a pulse of 106 beats/minute, a blood pressure of 80/60 mmHg, along with cool extremities and raised venous lactate (5.7 mEq/L), suggesting shock. Per-abdominal examination revealed a diffusely tender abdomen, and hence, a contrast-enhanced CT of her abdomen was ordered, which showed edematous changes in the walls of the terminal ileum, ileocecal valve, cecum, and large bowel, suggesting an infectious process (Figure 1).

Preliminary laboratory investigations, however, suggested only moderate anemia (hemoglobin of 9.7 g/dL). Blood, urine, and stool cultures were sent, following which she was started on empirical intravenous ceftriaxone, metronidazole, and inotropic support. Despite these measures, her clinical status worsened as indicated by worsening leukopenia (total leukocyte count of 2,100/μL) associated with relative lymphocytosis (absolute lymphocyte count of 1,239/μL). Empirical antibiotic therapy was escalated to meropenem. Although urine and stool cultures were sterile, Salmonella Paratyphi B was isolated on blood culture. Ceftriaxone sensitivity was confirmed, meropenem was withdrawn, and targeted antimicrobial therapy was instituted with intravenous ceftriaxone at a dose of 2 g twice daily. The patient showed a good response, with her laboratory parameters indicating immune reconstitution (total leukocyte count of 9,300/μL) and complete clinical resolution. The laboratory parameters of all three cases are summarized in Table 1.

**Table 1:** Summary of laboratory findings

Laboratory investigations	Case 1	Case 2	Case 3	Reference range
Hemogram				
Hemoglobin (g/dL)	13.6	13.0	9.7	13.0-17.0
Total leukocyte count (per μL)	13,400	8,500	7,600	4,000-11,000
Platelet count (per mm^3^)	155,000	26,000	184,000	150,000-450,000
Markers of inflammation and sepsis				
Procalcitonin (ng/mL)	8.66	-	-	<0.1
Renal function tests				
Serum creatinine (mg/dL)	4.33	0.6	1.58	0.55-1.02
Liver function tests				
Total bilirubin (mg/dL)	0.9	8.8	0.8	0.3-1.2
Direct bilirubin (mg/dL)	0.4	7.04	0.5	0-0.5
Indirect bilirubin (mg/dL)	0.5	1.76	0.4	0.2-0.7
Aspartate transaminase (U/L)	70	141	35	<35
Alanine transaminase (U/L)	35	48	25	<45
Serum alkaline phosphatase (U/L)	60	252	61	40-150
Cultures				
Blood	Salmonella Paratyphi B	Salmonella Typhi	Salmonella Paratyphi B	-
Stool	Salmonella Paratyphi B	-	No growth	-
Urine	No growth	-	No growth	-

## Discussion

The present series summarizes the heterogeneous manifestations of enteric fever beyond classic gastrointestinal symptoms.

The first case in our series was a case of Salmonella-induced septic shock complicated by sequential tachyarrhythmias. Cardiac complications like pericarditis, myocarditis, endocarditis, congestive heart failure, and ECG changes are reported in approximately 5% cases [6]. While isolated ECG changes may occur in 40%-80% cases, pathological ECG changes classically involve QT-prolongations, ST or T-wave deviations, bundle branch block, first-degree A-V block, and arrhythmias [7]. These changes are usually attributed to toxin-mediated myocarditis or electrolyte disturbances. A parallel line of thought argues that these changes may occur secondary to sepsis. However, such changes do not commonly occur in patients with normal coronary arteries. Furthermore, in enteric fever, such changes occur late in the disease and even occur in the absence of cardiovascular risk factors, as was the scenario with our first case [8]. Our first patient had neither structural heart disease nor electrolyte imbalance, suggesting a probable cytokine-induced arrhythmogenic mechanism, echoing observations from other systemic infections [9]. While arrhythmias such as the Wenckebach phenomenon, complete heart block, and ventricular bigeminy have been described in cases of enteric fever, the present series is the first one to document the occurrence of sequential tachyarrhythmias consisting of two episodes of paroxysmal supraventricular tachycardia followed by an episode of sustained atrial fibrillation, requiring chemical cardioversion, in the setting of enteric fever [10-12].

The second case was one of enteric fever presenting with icterus and early isolated thrombocytopenia. Hepatic involvement encountered with enteric fever is typically mild and more often manifests as mildly deranged liver function tests and hepatomegaly [13]. Icterus is a relatively rare occurrence in enteric fever, with an incidence ranging from 4.8% to 17.6% of cases [14]. Furthermore, enteric fever presenting with icterus usually correlates with the biochemical finding of conjugated hyperbilirubinemia and a mild rise in transaminase enzymes. This finding further supports the hypothesis that a predominant hepatic inflammatory pathology, resulting in biliary canaliculi occlusion by swollen hepatocytes, drives the development of icterus rather than a hemolytic process [15]. Interestingly, while our patient also presented with conjugated hyperbilirubinemia, with an almost negligible contribution from the indirect bilirubin pool and mild transaminitis suggesting hepatic inflammation, his laboratory profile was also significant for severe thrombocytopenia. The occurrence of thrombocytopenia is an uncommon manifestation in enteric fever and can lead to diagnostic confusion with viral or leptospiral hepatitis, especially when accompanied by icterus [16]. It can occur transiently in 10%-15% cases, after the first week of illness. While the exact mechanism by which thrombocytopenia occurs is poorly understood, its persistence is typically associated with a more fulminant form of the disease. While a negative serology may further exacerbate diagnostic delays, persistent fever despite empirical antibiotic therapy should prompt clinicians to consider enteric fever in endemic regions.

The third and final case in our series was a case of Salmonella enterocolitis-associated sepsis complicated by leukopenia. Leukopenia, secondary to systemic salmonellosis, is a prominent but nonuniversal feature encountered in 20%-25% cases of enteric fever. Its occurrence, just like thrombocytopenia, is attributed to the invasion of hematopoietic organs by Salmonella, leading to marrow suppression and hemophagocytosis [17]. Targeted antibiotic therapy and careful monitoring of marrow function are essential while awaiting immune reconstitution in such cases. It is worth mentioning that derangements in blood parameters, such as leukopenia and thrombocytopenia, offer a more negative predictive value in suspected tropical illnesses, helping to differentiate between viral and bacterial fevers.

Another biochemical finding in our third and first cases was elevated levels of serum creatinine, suggesting AKI. The degree of serum creatinine elevation in the third case, who did present with diarrhea and shock, was relatively mild (1.55 mg/dL). In contrast, our first case, while lacking a presentation significant for diarrhea, did develop septic shock and a greater elevation of serum creatinine levels (4.33 mg/dL). Renal involvement in enteric fever is quite rare (2%-3% cases) and is frequently attributed to dehydration, shock, and, rarely, rhabdomyolysis [18]. Although the causes of AKI in many cases of enteric fever are still uncertain and a multifactorial etiology cannot be ruled out, the most likely explanation for the development of AKI in our series is likely dehydration and shock, producing a prerenal form of AKI. The assumption is justified based on the return to baseline of serum creatinine levels of both cases in response to adequate intravenous hydration and resolution of sepsis.

The present series reinforces that clinicians, in endemic areas, must maintain a high index of suspicion for enteric fever, initiate empirical broad-spectrum antibiotics, and refine therapy based on culture results. The series also highlights the utility of bedside and cost-effective imaging techniques such as POCUS for the rapid evaluation of undifferentiated sepsis. Specifically with regard to the second case in our series, the finding of pseudothickening of the gallbladder wall alerted the authors to the possibility of enteric fever, in view of the organism's preference for colonizing the same [19]. Furthermore, the present series highlights that while modern, multiplex molecular assays allow for rapid identification of infectious agents, conventional microbiological diagnostic tests, such as cultures, remain the gold standard for diagnosing enteric fever. Although newer modalities such as metagenomic next-generation sequencing (mNGS) are found to be highly reliable and capable of detecting other features of potential clinical interest beyond pathogen identification, the lack of approved methods, instruments, and/or databases for diagnostic mNGS currently limits widespread adoption by the medical community [20]. Future avenues of research in molecular diagnosis of enteric fever should be directed at extensive validation and demonstration of performance metrics, to enable widespread adoption of mNGS.

## Conclusions

The current series aims to highlight unusual presenting features and complications of enteric fever. The symptom and laboratory profile of enteric fever shows considerable overlap with other tropical illnesses. From a practical point of view, clinicians must be aware and remain vigilant for such uncommon presentations. Adopting an individualized, case-by-case approach, even when preliminary serological tests are negative, will enable clinicians to arrive at a diagnosis without delaying treatment. Ensuring timely cultures and empirical antibiotic therapy while awaiting microbiological confirmation will allow clinicians to optimize clinical outcomes and thereby reduce enteric fever-related morbidity and mortality.
